# Assessing the efficacy of the natural disaccharide trehalose in ameliorating diet-induced obesity and metabolic dysfunction

**DOI:** 10.3389/fnut.2025.1580684

**Published:** 2025-06-02

**Authors:** Yu-Sheng Yeh, Trent D. Evans, Se-Jin Jeong, Ziyang Liu, Ali Ajam, Carlos Cosme, Jun Huang, Doureradjou Peroumal, Xiangyu Zhang, Ali Javaheri, Jaehyung Cho, Irfan J. Lodhi, Babak Razani

**Affiliations:** ^1^Department of Medicine, Vascular Medicine Institute, University of Pittsburgh School of Medicine and UPMC, Pittsburgh, PA, United States; ^2^Pittsburgh VA Medical Center, Pittsburgh, PA, United States; ^3^Cardiovascular Division, Department of Medicine, Washington University School of Medicine, St. Louis, MO, United States; ^4^Division of Hematology, Department of Medicine, Washington University School of Medicine, St. Louis, MO, United States; ^5^Division of Endocrinology, Metabolism, and Lipid Research, Department of Medicine, Washington University School of Medicine, St. Louis, MO, United States

**Keywords:** trehalose, obesity, insulin resistance, hepatosteatosis, trehalase, oral vs. parenteral

## Abstract

Trehalose is a naturally occurring disaccharide with versatile commercial applications and health benefits, including promise as a therapeutic for obesity and diabetes. Although numerous previous reports purport the therapeutic uses of orally ingested trehalose, the abundance of glycosidases in the gastrointestinal tract suggest the potential for significant limitations of oral trehalose that have not been addressed. We first fed mice a high-fat diet (HFD) while providing trehalose by both oral and intraperitoneal routes. This combined strategy was broadly efficacious in reversing HFD-induced weight gain, fat mass, insulin resistance, and the development of hepatosteatosis. In contrast, oral-only trehalose failed to improve HFD-induced obesity and insulin resistance. This was due to trehalase (Treh)-mediated metabolism as blood trehalose levels remained low despite a significant rise in glucose. We next developed systemically deficient Trehalase (Treh-KO) mice to enhance the efficacy of trehalose. Surprisingly, oral trehalose therapy could not be facilitated resulting in neither an increase in serum trehalose levels nor metabolic benefits. Parenteral trehalose resulted in higher trehalose levels with lower serum glucose in Treh-KO mice, yet no additive metabolic benefits were observed. Overall, our findings still support a therapeutic role for trehalose in obesity and metabolic disease but with practical limitations in its delivery by oral route.

## Introduction

Western diets and increasingly sedentary lifestyles have contributed significantly to the growing rate of obesity over the last several decades (1). Obesity is simply defined as the over-accumulation of fat in white adipose tissue (WAT) due to an increase in the ratio of energy intake to energy expenditure (2). However, accumulating evidence has shown that adipose tissue is not a simple, dormant site of energy storage but a dynamic metabolic organ that secretes a large number of factors, such as lipids and cytokines (i.e., adipokines), with hormonal, autocrine, and paracrine properties (3, 4). Adipose tissue is therefore thought to actively participate in the modulation of systemic metabolic homeostasis, and adipose tissue dysfunction is believed to be a major culprit for obesity-related metabolic diseases, such as insulin resistance and fatty liver disease (3).

Dietary and lifestyle changes such as consuming balanced diet low in saturated fats and high in complex carbohydrates are considered the most effective means of reducing obesity, yet obesity remains a major health epidemic, especially when considering its burgeoning comorbidities in conjunction. There are various anti-obesity medications available including the popular GLP-1 receptor agonists which are appetite suppressants leading to reduced caloric intake, but these are accompanied by adverse side effects which can curtail their long-term use (5–7). Natural anti-obesity agents are always an attractive alternative which raise the prospect of trehalose with several proposed benefits in obesity and the metabolic syndrome.

Trehalose is a disaccharide produced naturally by a wide variety of plants, insects, and microorganisms such as bacteria, yeast, and fungi, and synthesized commercially as a sweetener and preservative (8). Trehalose is composed of two glucose molecules linked by a unique *α*,α-1,1-glycosidic bond which provides it exceptional stability under a wide range of conditions, including high temperatures, low pH, and freezing temperatures. It is notable that in nature trehalose acts as a protective agent against stress, allowing microorganisms to survive in adverse conditions, such as desiccation, heat, or cold. Although trehalose is not able to be synthesized in vertebrates (9), accumulating data indicate that it acts as a functional molecular compound to modulate metabolic and cellular processes in mammals, including humans. The abundance of trehalase in the GI tract allows its enzymatic hydrolysis into glucose as an obvious source of carbon. However, in an intact form, its structural properties might contribute to the ability of trehalose to alter the gut microbiome (10) and alter glucose homeostasis (11).

Much of the excitement for the therapeutic impact of trehalose in various chronic diseases lies in its ability to induce autophagy and in turn reduce the burden of cytotoxic protein aggregates. Trehalose has been proposed as a therapy for neurodegenerative diseases (12) with a significant burden of protein aggregation such as Parkinson’s Disease (13), Alzheimer’s Disease (14), and Huntington Disease (15), as well as cardiovascular disease, including atherosclerosis (16, 17), and hypertension (18). Although the mechanisms remain unclear including whether autophagy is primarily involved, several studies have shown that trehalose also improves metabolic health including dyslipidemia, obesity, diabetes, and fatty liver disease. For example, oral trehalose treatment reverses plasma insulin levels and homeostasis model assessment-insulin resistance (HOMA-IR) in mice fed a high-fat diet (HFD) (19), whereas intraperitoneal injection (i.p.) ameliorates insulin resistance in ob/ob mice (20). Similar results were obtained using a mouse model of established obesity (21). Reduced adipocyte size with enhanced thermogenic brown fat markers in WAT have also been reported (19, 21, 22). Trehalose treatment also improves hepatic steatosis including reduced accumulation of lipid droplets in hepatocytes (23). Similarly, db/db mice treated with trehalose exhibited decreased lipid inclusions in the liver accompanied by an increase in glycogen content (24). Trehalose supplementation has been shown to reduce hepatic endoplasmic reticulum stress and inflammatory signaling in aged mice (25). Collectively, these studies strongly support trehalose as a therapeutic candidate for the prevention and possible reversal of obesity-associated metabolic disorders.

An outstanding issue in the practical therapeutic use of trehalose in metabolic disease is the lack of consensus regarding trehalose dosage, administration route, or treatment time course. The presence of trehalase in the GI tract would suggest that absorption of trehalose should be compromised, albeit many studies report positive findings with oral-only trehalose formulations without taking into account confounding effects on microbiota or osmotic effects in the GI tract. In the current study, we aimed to definitively study the efficacy of trehalose in obesity and metabolic disease while taking into account both the route of administration and the oft neglected role for trehalase. We develop and characterize Trehalase-deficient mice (Treh-KO) both at baseline and in the context of trehalose under HFD condition. Our findings that an oral route of trehalose administration as well as inhibition of trehalase are ineffective in mitigating diet-induced obesity and metabolic disease should provide clarity for the field.

## Results

### Trehalose treatment reduces high fat diet-induced obesity

**Figure 1:**
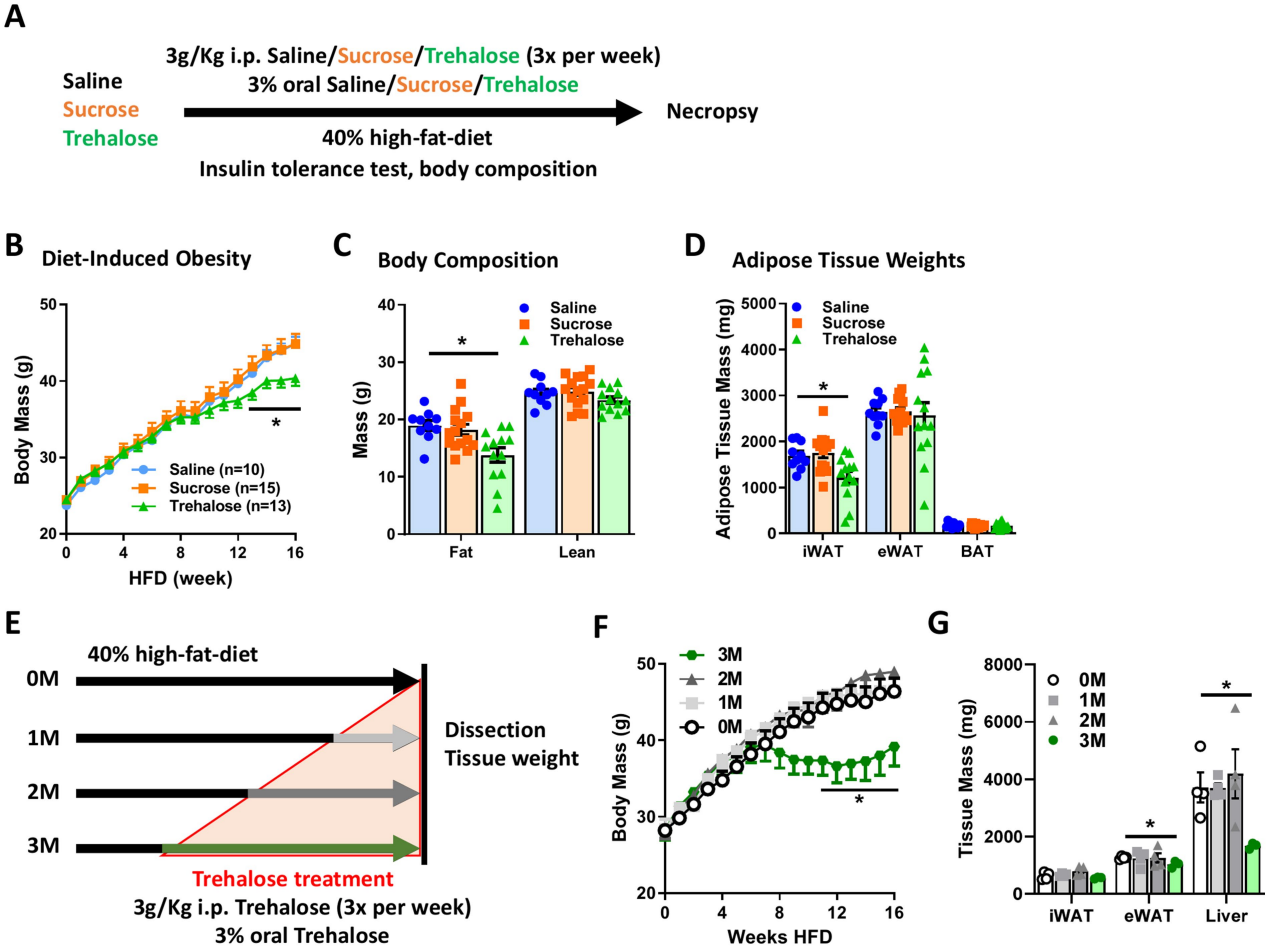
Combination of oral and intraperitoneal trehalose treatment abrogates high-fat diet-induced obesity. **(A)** Schematic process of trehalose or sucrose intervention experiment. The mice were treated with saline, sucrose, or trehalose by injecting intraperitoneally (3 times per week) and as a supplement to drinking water. **(B)** Body weight gain, **(C)** body composition, and **(D)** iWAT, eWAT, and BAT weights of mice treated with saline (*n* = 10), sucrose (*n* = 15), or trehalose (*n* = 13) under HFD condition were measured after 16 weeks of HFD feeding. **(E)** Schematic illustration of trehalose treatment time course experiment. The mice were fed HFD for 16 weeks, during which time mice also received trehalose for 0, 1, 2, or 3 months (0 M, 1 M, 2 M or 3 M) both orally and intraperitoneally. **(F)** Body weight variation, and **(G)** Tissue mass of iWAT, eWAT, and liver in the mice after 16 weeks of HFD treatment, along with trehalose added to drinking water for 0 M, 1 M, 2 M, and 3 M (*n* = 4). All mice were male and fed HFD. Values are presented as mean ± SE. Significant differences were determined by Student’s *t*-test, with comparison to Saline or 0 M group: *p < 0.05.

We previously demonstrated that the combination of oral and intraperitoneal (i.p.) administration of trehalose is atheroprotective in mice fed a western diet (40% high-fat diet, HFD) (26). To investigate the effects of trehalose on obesity and other metabolic parameters, 8-week-old C57BL/6 J mice fed a HFD received i.p. administration (3 g/Kg body weight) of trehalose 3 times per week, along with drinking water supplemented with 3% trehalose. Control mice received the equivalent co-administered doses of either saline or sucrose, a similarly structured non-reducing disaccharide composed of glucose and fructose (Figure 1A). HFD-induced body weight gain was significantly suppressed after 12–16 weeks of trehalose administration, in contrast to the sucrose-treated group, which was indistinguishable from the saline control (Figure 1B). Additionally, the trehalose group showed an overall significantly lower fat mass compared to controls without a reduction in lean mass (Figure 1C). Inguinal WAT (iWAT) weight was similarly reduced only in trehalose treated mice (Figure 1D). There were no changes in body weight, fat mass, and adipose tissue weight with saline or sucrose treatments for the same time course (Figures 1B–D).

To examine functional duration and efficacy of treatment, mice were fed a HFD for 4 months (4 M) and co-administrated trehalose by i.p. (3 g/Kg body weight) and in drinking water (3%) 3 times per week for a total treatment time of 3 M, 2 M, or 1 M after HFD feeding as compared with an un-treated HFD group denoted as 0 M (Figure 1E). The trehalose 3 M-treated group, but not 1 M and 2 M groups, exhibited a dramatic decrease in HFD-induced body weight gain (Figure 1F). Although no significance was noticed in the iWAT weight, the epididymal WAT (eWAT) weight was significantly reduced in the 3 M group, compared to that in the 0 M control group (Figure 1G), indicating an effective intervention period requires sustained exposure to trehalose.

### Trehalose ameliorates high fat diet-induced insulin resistance and hepatic lipid accumulation

**Figure 2:**
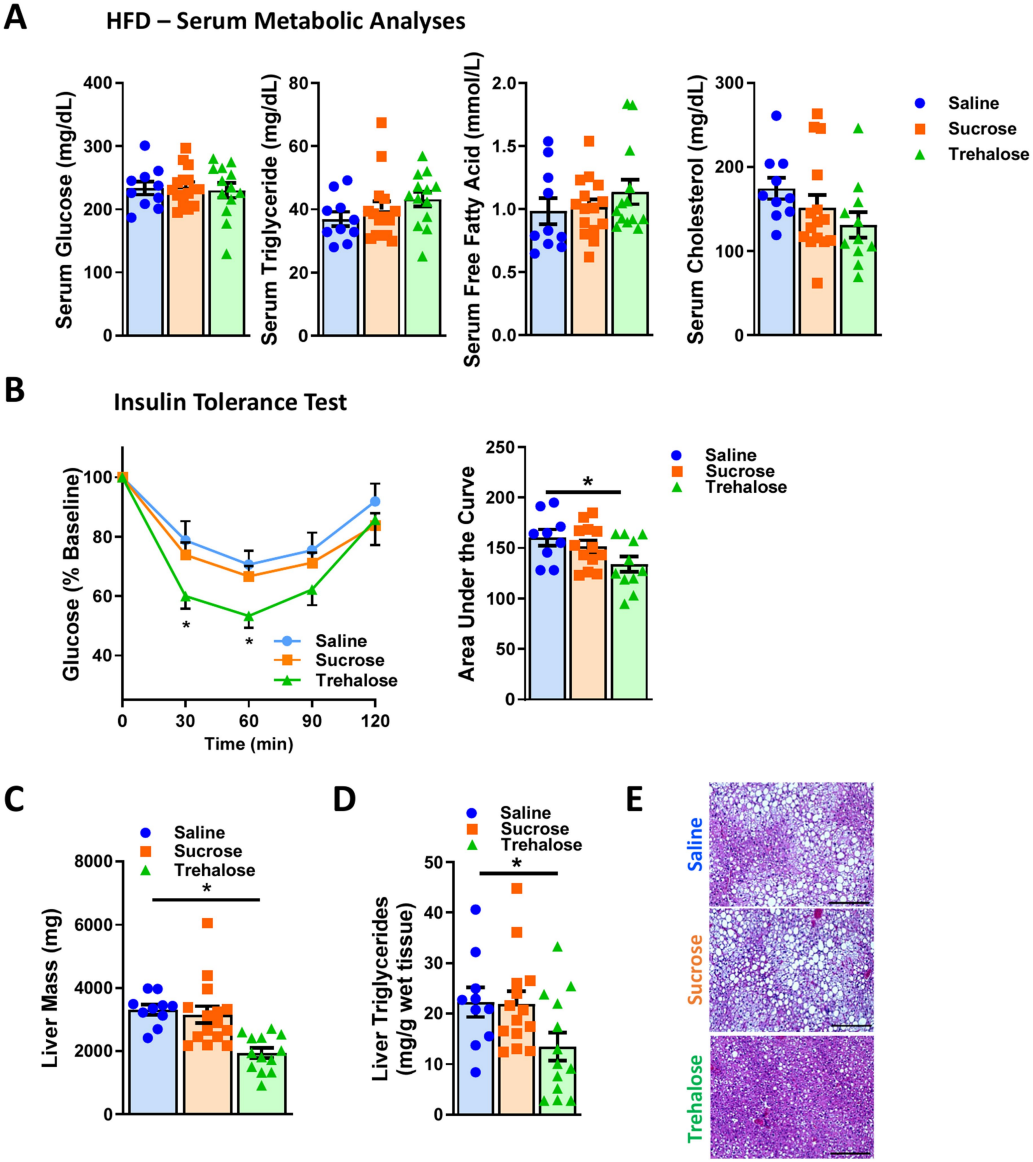
Combination of oral and intraperitoneal trehalose treatment improved high-fat diet-induced insulin resistance and fatty liver. **(A)** Serum glucose, triglyceride, free fatty acid, and cholesterol levels of mice receiving saline (*n* = 10), sucrose (*n* = 15), or trehalose (*n* = 13) were measured at 16 weeks of HFD feeding. **(B)** Insulin tolerance test was performed at 15 weeks of HFD-fed mice treated with saline (*n* = 10), sucrose (*n* = 15), or trehalose (*n* = 13). Results were shown as a time-dependent graph (left panel) or as the area (%*min) under the curve (right panel). **(C)** Liver mass, **(D)** triglyceride content, and **(E)** histological analysis with H&E staining were performed. Liver samples from 16 weeks HFD-fed mice treated with saline (*n* = 10), sucrose (*n* = 15), or trehalose (*n* = 13). Scale bars, 200 μm. All mice were male and fed HFD. Values are presented as mean ± SE. Significant differences were determined by Student’s *t*-test, as compared with Saline group: *p < 0.05.

To investigate whether trehalose modulates HFD-associated metabolic parameters, we measured the levels of serum glucose, triglyceride, free fatty acid, and cholesterol after co-administration of trehalose for 4 M. Although trehalose reduced HFD-induced weight gain (Figures 1B–D), no significant changes were found in the evaluated serum metabolites (Figure 2A). However, insulin tolerance testing (ITT) revealed significantly decreased blood glucose levels at 30 and 60 min after insulin administration in the trehalose group, compared with saline and sucrose control groups (Figure 2B), suggesting enhanced insulin sensitivity with trehalose treatment concomitant with the observed reductions in body weight and adiposity.

We also noticed a significant decrease in the liver weight in the trehalose group (Figure 2C). Given obesity and adipose tissue dysfunction lead to ectopic lipid accumulation in lean organs such as liver (27), we suspected the effect of trehalose on the liver might be due to lower hepatic lipid accumulation. To confirm this, the degree of hepatic lipid accumulation was quantified biochemically and by histological analysis. As shown in Figure 2D, trehalose treatment reduced hepatic lipid content by ~40%, compared with saline or sucrose treatments. Similarly, there were marked reductions in lipid droplet accumulation in hematoxylin and eosin (H&E)-stained liver sections from trehalose-treated mice (Figure 2E), indicating the ability of trehalose to reduce overall hepatic lipid burden instigated by HFD feeding. It should be noted that trehalose treatment did not result in significant changes in the expression of genes related to either adipose tissue browning, mitochondrial lipid metabolism, or the autophagy-lysosomal system (Supplementary Figure S1). Although transcriptional effects were not noted to be relevant to trehalose function, this does not preclude direct effects on autophagy and the lysosomal system as has been previously reported (16, 28).

### Oral trehalose is therapeutically ineffective in ameliorating obesity and insulin resistance

**Figure 3:**
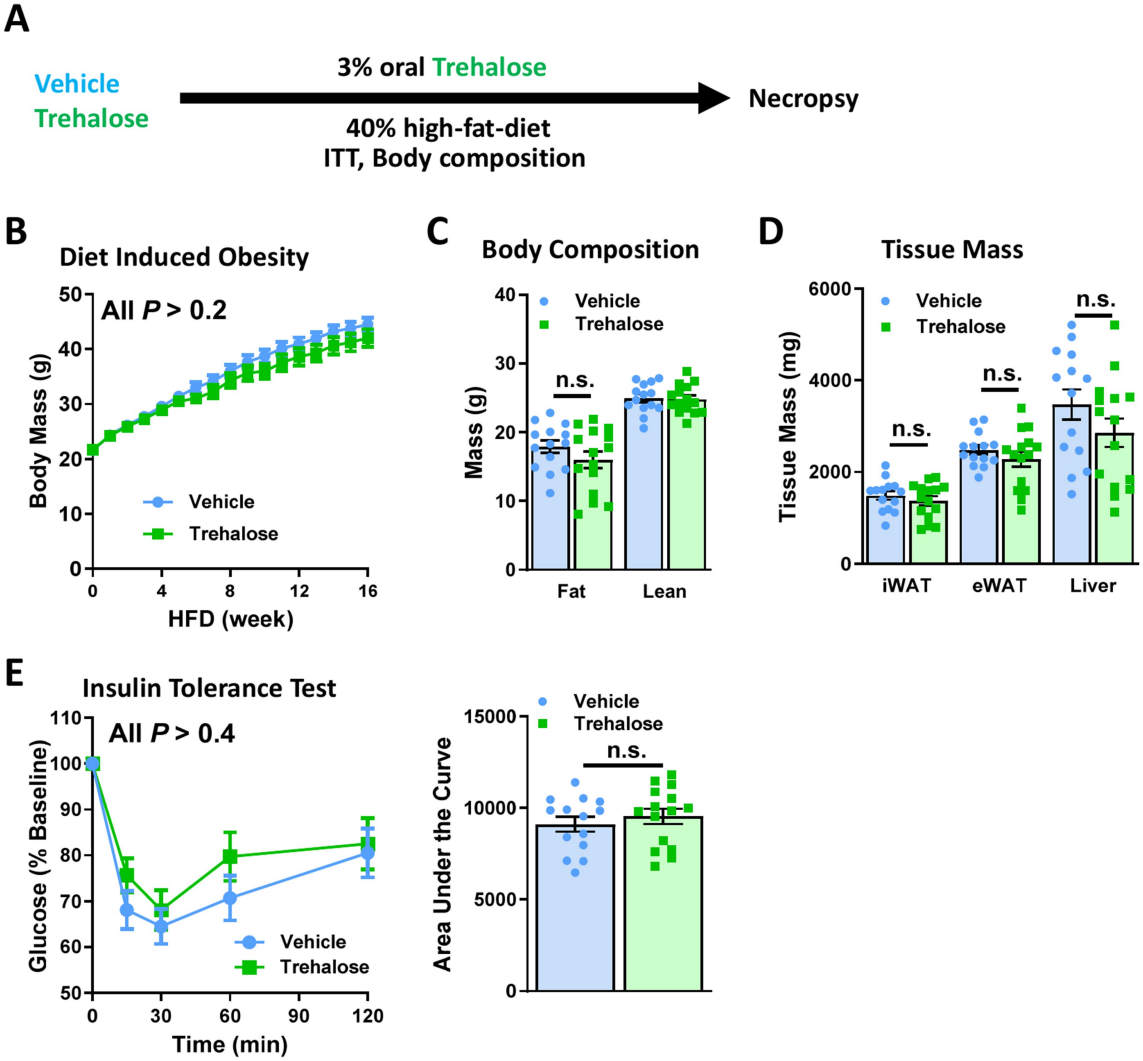
Oral trehalose is therapeutically ineffective. **(A)** Schematic representation of trehalose or vehicle intervention experiment. Treatments were provided as supplementation to drinking water. **(B)** Body weights were measured in mice provided drinking water with or without 3% w/v trehalose (*n* = 15). **(C)** Body composition, and **(D)** tissue weights of iWAT, eWAT, and liver were measured after 16 weeks of HFD feeding with or without 3% w/v trehalose as a supplement in drinking water (*n* = 15). **(E)** Insulin tolerance test was performed at 15 weeks HFD-fed mice treated with or without 3% w/v trehalose, provided as a supplement in drinking water (*n* = 15). Results were shown as a time-dependent graph (left panel) or as the area (%*min) under the curve (right panel). All mice were male and fed HFD. Values are presented as mean ± SE.

Although oral-only and i.p.-only trehalose treatments have been independently reported to have metabolic benefits (20, 21), relative contributions of each to its metabolic efficacy remain unknown. We thus examined each trehalose treatment route individually, beginning with oral-only administration in drinking water (3% w/v) with HFD for 16 weeks (Figure 3A). Body weight gain was slightly lower in oral trehalose-treated mice, but this difference was insignificant compared with control animals (Figure 3B). Likewise, there was no change in body composition (Figure 3C), nor in tissue mass including iWAT, eWAT, or liver between controls and the oral trehalose-treated group (Figure 3D). Oral trehalose treatment also had no effect on insulin sensitivity as gaged by similar glucose levels after an ITT assay (Figure 3E).

### Oral trehalose treatment has negligible effects on circulating trehalose levels

**Figure 4:**
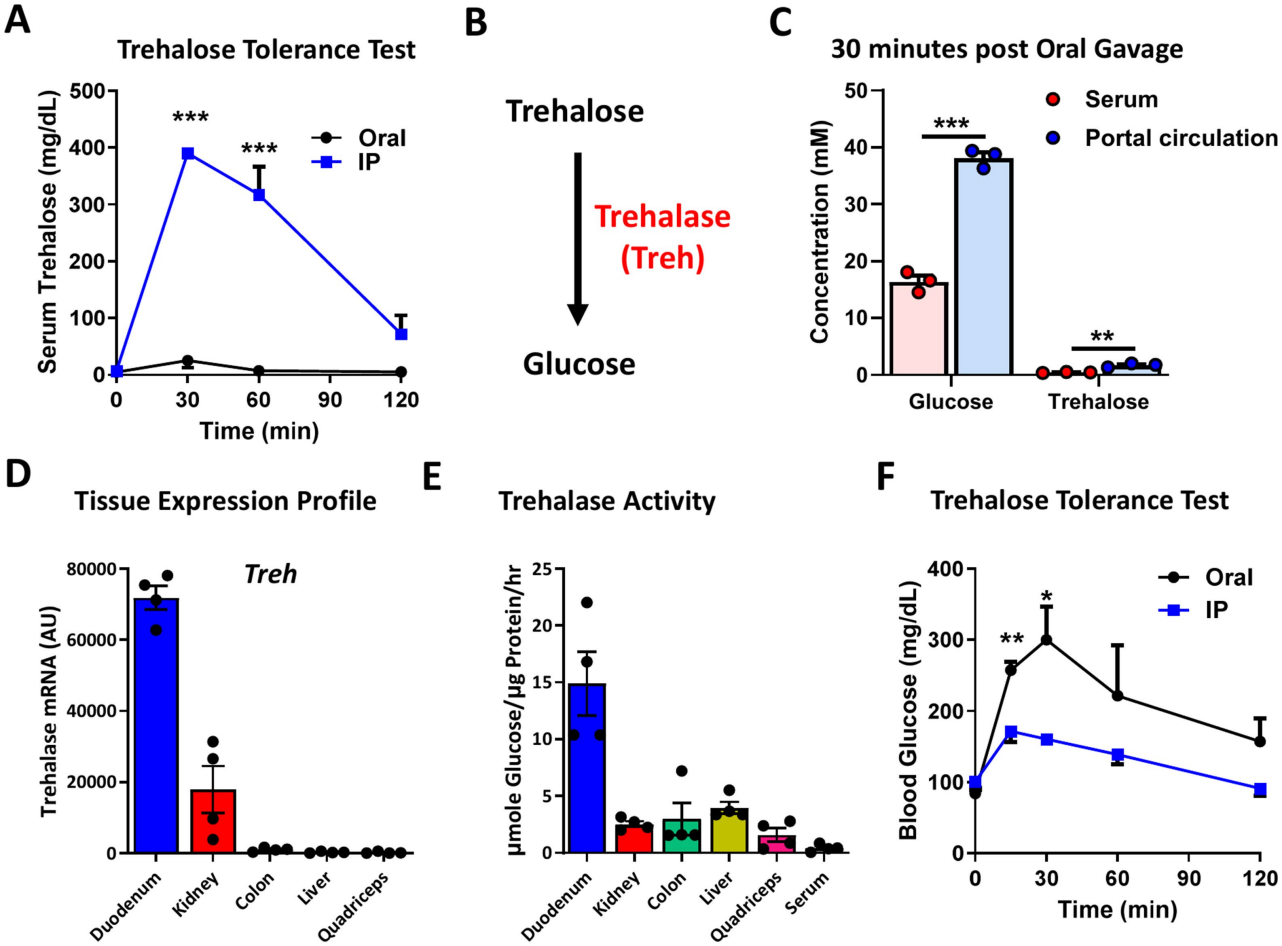
Oral trehalose treatment enhanced blood glucose level but only show a minor effect on serum trehalose. **(A)** Trehalose tolerance test was performed based on oral (Oral) and intraperitoneal (IP) treatment (3 g/Kg body weight) and the serum trehalose level was measured at indicated time points (*n* = 4). **(B)** Schematic image of the metabolism of trehalose through trehalose (Treh) to glucose. **(C)** Glucose and trehalose levels were measured in serum and portal circulation 30 min after oral trehalose treatment (*n* = 3). **(D)** Tissue mRNA expression and **(E)** activity of Treh were measured in the duodenum, kidney, colon, liver, and quadriceps of 8-week-old male C57BL/6 J mice (*n* = 4). **(F)** Trehalose tolerance test was performed based on oral (Oral) and intraperitoneal (IP) treatment (3 g/Kg body weight) and the serum glucose level was measured at indicated time points (*n* = 4). All mice were male and fed a normal chow diet. Values are presented as mean ± SE. Significant differences were determined by Student’s *t*-test compared with IP group: *p < 0.05, **p < 0.01, ***p < 0.001.

To ascertain whether the lack of effectiveness for oral trehalose against obesity and metabolic dysfunction is due to low absorption or high catabolic rate, we performed a trehalose tolerance test to compare blood trehalose levels between oral and i.p. trehalose administration. Interestingly, serum trehalose was enhanced to ~400 mg/dL at 30 min after i.p. trehalose administration and decreased gradually in a time-dependent manner (Figure 4A). However, oral trehalose failed to elevate serum trehalose levels (Figure 4A), suggesting that trehalose does not enter the bloodstream, possibly because it is hydrolyzed by trehalase (Treh) into its two component glucose molecules in the gastrointestinal tract (Figure 4B). This was corroborated by portal vein sampling in conjunction with assessment of serum glucose and trehalose levels upon oral trehalose administration. Whereas serum glucose rises significantly after 30 min of trehalose ingestion, a minimal rise in portal vein levels of trehalose is observed indicating near complete trehalose hydrolysis in the intestine (Figure 4C).

To determine the plausibility of these two possibilities, we next measured expression and activity levels of Treh in several tissues, including the duodenum (the absorptive portion of the intestine). We found that Treh was highly expressed in the duodenum and kidney, but was not detectable in colon, liver, or quadriceps (Figure 4D). The duodenum also had the highest Treh enzymatic activity among the evaluated tissues (Figure 4E), consistent with the possibility that higher catabolism of trehalose in the intestine accounts for lower observed blood levels of trehalose. To determine whether trehalose might indeed be degraded in orally treated mice, blood glucose levels were monitored during the trehalose tolerance test. In contrast to mice administered trehalose by i.p., which had only 1.7-fold increase in blood glucose levels relative to baseline, glucose increased 3.6-fold in mice treated orally with trehalose (Figure 4F), indicating more than twice as much enzymatic digestion prior to absorption.

### Mice with systemic trehalase deficiency have not metabolic defects at baseline

**Figure 5:**
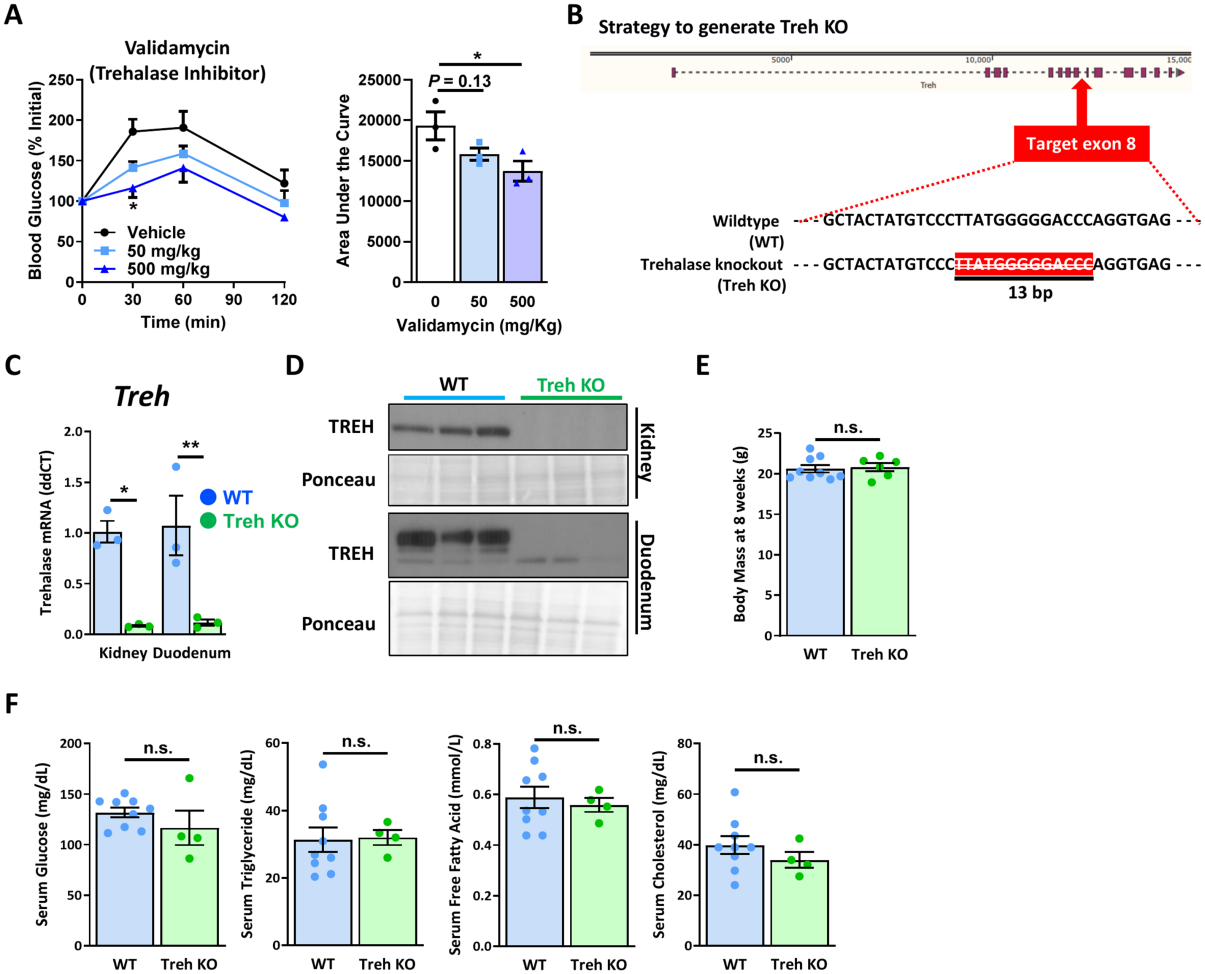
Systemic trehalase-deficient mice showed no metabolic changes at baseline. **(A)** Indicated dose of validamycin (Trehehalase inhibitor) was used for mice treated with trehalose (3 g/Kg body weight), after 6 h fasting. Blood glucose levels were measured at indicated time points (left panel), and area under the curve (%*min) was calculated (right panel) (*n* = 3). **(B)** Illustration of strategy used to establish a systemic trehalase (Treh) knockout (Treh KO) mouse model. Treh DNA genotyping was performed on mouse tail clippings from heterozygous (Het), wildtype (WT) and Treh KO mice (Embedded panel). **(C)** mRNA and **(D)** protein expression levels of Treh in the kidney and duodenum of 8-week-old WT and Treh KO male mice (*n* = 3). **(E)** Body weight, and **(F)** serum glucose, triglyceride, free fatty acid, and cholesterol levels were measured in 8-week-old female WT (*n* = 9) and Treh KO (*n* = 6) mice. All mice were fed a normal chow diet. Values are presented as mean ± SE. Significant differences were determined by Student’s *t*-test in comparison with vehicle or WT group: *p < 0.05, **p < 0.01.

To determine whether suppression of trehalose catabolism could be a useful approach of maintaining elevated trehalose levels, a trehalose tolerance test was performed after pretreatment with Validamycin, a known Treh inhibitor (29). Enhancement of blood glucose levels in response to oral trehalose treatment was partially suppressed in the presence of Validamycin in a dose-dependent manner (Figure 5A), suggesting the utility of sustained Treh inhibition in achieving enhanced uptake and efficacy of trehalose *in vivo*.

We therefore engineered a mouse model with Treh deficiency via CRISPR/Cas9 (Figure 5B) by removal of a 13 bp region within exon 8 of the Treh gene (Figure 5B). To confirm whether Treh expression was successfully disrupted in homozygous Treh knockout (KO) mice, transcriptional and protein expression of Treh in kidney and duodenum were measured by qPCR and Western blotting, respectively. As shown in Figure 5C, mRNA levels of *Treh* were suppressed ~90% in Treh KO kidney and duodenum tissues, compared with those from the wildtype (WT) group. Protein levels of Treh in the KO group were likewise nearly undetectable in both tissues (Figure 5D), suggesting that a systemic Treh-disrupted mouse model was successfully established. In 8-week-old adult mice, no body weight differences were noted (Figure 5E). Serum glucose, triglyceride, free fatty acid, and cholesterol levels were also similar between WT and Treh KO groups (Figure 5F).

### Systemic trehalase deficiency does not improve the negligible rise of circulating trehalose in mice treated by oral administration

**Figure 6:**
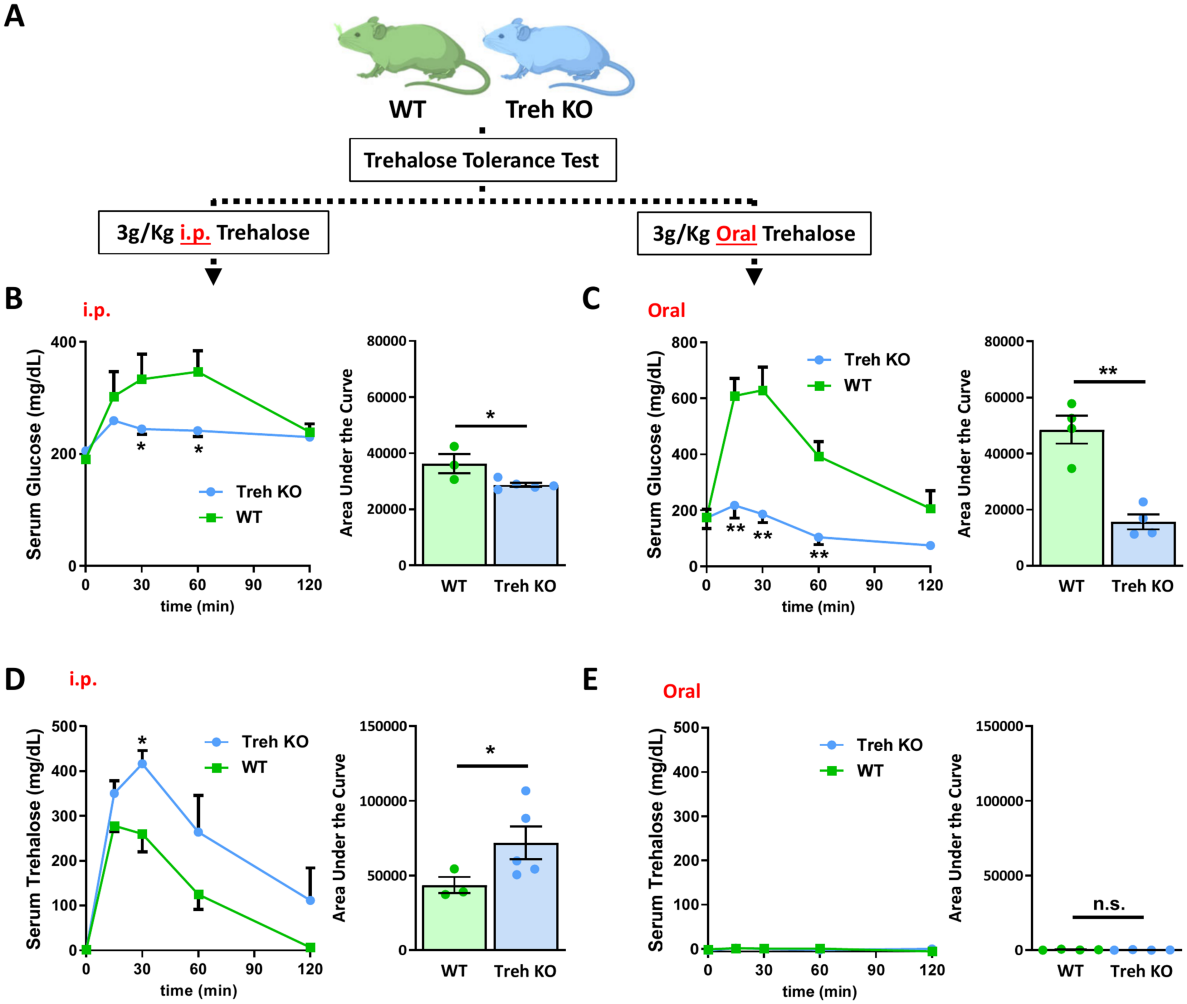
Systemic trehalase deficiency delayed serum trehalose clearance but did not improve trehalose oral absorption. **(A)** Schematic illustration of the experimental process employed. WT and Treh KO mice were tested for trehalose tolerance test after oral or i.p. trehalose administration. Serum glucose levels were quantified at indicated time points (left panel) after **(B)** i.p. (WT and Treh KO, *n* = 3 and 5) or **(C)** oral (*n* = 4) trehalose treatment (3 g/Kg body weight) and area under the curve calculated (mg/dL*min, right panel). Serum trehalose levels were quantified at indicated time points (left panel) after **(D)** i.p. (WT and Treh KO, *n* = 3 and 5) or **(E)** oral (*n* = 4) trehalose treatment (3 g/Kg body weight), and area under the curve calculated (mg/dL*min, right panel). All mice were male and fed a normal chow diet. Values are presented as mean ± SE. Significant differences were determined by Student’s *t*-test, as compared with WT group: *p < 0.05, **p < 0.01.

To determine the effect of trehalase deficiency on the kinetics of serum trehalose and glucose, Treh KO mice and WT controls were administered trehalose either by i.p. (3 g/kg) or oral routes (3 g/kg) and trehalose tolerance tests performed (Figure 6A). Similar to the results shown in Figure 4F, i.p. trehalose treatment increased serum glucose levels 1.8-fold in the WT group but only 1.25-fold in Treh KO mice (Figure 6B). In orally treated WT mice, serum glucose levels were enhanced more than 3-fold, while only a minor rise was observed in the Treh KO group (Figure 6C). With regard to serum trehalose levels, the contrasts between the oral and parenteral routes were highly revealing. Serum trehalose levels were substantially increased in WT mice administered trehalose by i.p., and this effect was increased even further in Treh KO animals (Figure 6D). Serum trehalose WT group reached peak levels at 15 min and returned nearly to baseline by 120 min, whereas levels in the Treh KO group reached a peak at 30 min followed by a gradual decrease (Figure 6D), suggesting that Treh deficiency was efficacious in reducing trehalose clearance. In contrast to the efficacy seen with i.p. trehalose, serum levels of trehalose in mice treated orally were only negligibly altered for both WT and Treh KO groups, suggesting trehalose hydrolysis is not responsible for poor oral absorption (Figure 6B).

### Systemic trehalase deficiency does not augment the metabolic benefits of parenteral trehalose in high fat diet-fed mice

**Figure 7:**
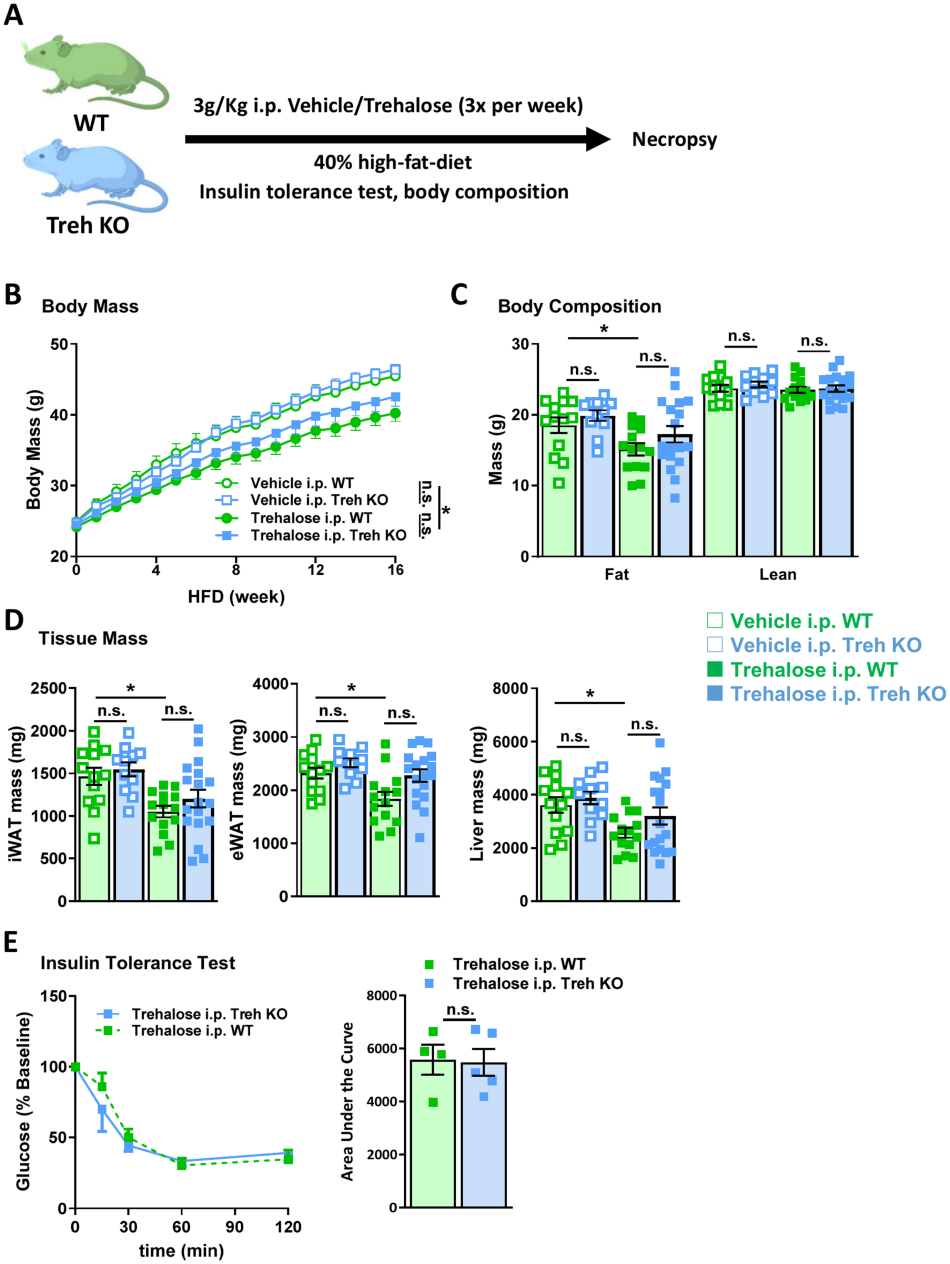
I.P. trehalose alone effectively reduced obesity and trehalase deficiency did not improve efficacy. **(A)** Schematic representation of trehalose or vehicle intervention experiment. WT and Treh KO mice were treated with vehicle or trehalose by i.p. injection. **(B)** Body weight, **(C)** body composition, and **(D)** tissue mass of iWAT (left panel), eWAT (middle panel), and liver (right panel) were measured in WT and Treh KO mice fed a HFD and treated by i.p. injection of trehalose or vehicle (3 g/Kg body weight, 3 times per week) for 16 weeks (Vehicle i.p. WT, Trehalose i.p. WT, Vehicle i.p. Treh KO, and Trehalose i.p. Treh KO, *n* = 13, 14, 11, and 18). **(E)** Insulin tolerance test was performed in WT (*n* = 4) and Treh KO (*n* = 5) mice i.p. injected with trehalose (3 g/Kg body weight, 3 times per week) for 15 weeks. Blood glucose level were measured at indicated time points (left panel), and area under the curve was calculated (mg/dL*min, right panel). All mice were male and fed HFD. Values are presented as mean ± SE. Significant differences were determined by Student’s *t*-test compared with indicated groups: *p < 0.05.

Given the blunted serum trehalose clearance observed in Treh KO mice administered trehalose by i.p., we wondered whether increased trehalose levels in the setting of Treh deficiency might lead to enhanced metabolic benefits. WT and Treh KO mice were fed HFD while injected with trehalose (3 g/Kg body weight i.p., 3x/week) or vehicle control (Figure 7A). After 16 weeks of diet with and without concomitant trehalose, there were no notable differences in body weight, fat mass, or tissue mass, including iWAT, eWAT, and liver, between WT and Treh KO groups (Figures 7B–D). However, the previously demonstrated trehalose-mediated reduction in HFD-induced body weight gain (Figure 1B) was replicated (Figure 7B). The HFD-induced increases in fat mass in WT mice were also significantly reduced in the Trehalose i.p. group, compared with the Vehicle control group (Figures 7C,D). Although i.p. administration of trehalose elicited a similar anti-obesity effect in Treh KO mice, there was no significant difference between WT and Treh KO mice based on treatment (Figures 7B–D). Likewise, similar effects on blood glucose were observed for both WT and Treh KO mice, as demonstrated by equivalent percent glucose decline relative to baseline after insulin tolerance testing (Figure 7E). Overall, these data suggest that parenteral treatment of trehalose is effective against HFD-induced obesity although no additive metabolic benefits are realized in the setting of elevated trehalose levels from Treh inhibition.

## Discussion

In the present study, we demonstrated that the putative anti-obesity disaccharide trehalose reduces body weight, fat mass, hepatic lipid accumulation, and insulin insensitivity in HFD-fed mice. Trehalose improved hepatic lipid accumulation with a decrease in liver weight, which is in line with previous studies showing positive effects of trehalose on hepatic lipid accumulation and stress (23–25). This evidence implies a possibility that the liver is the primary organ affected by trehalose, either directly or indirectly, which requires further study. We previously observed protection from atherosclerosis in mice via dual oral and i.p. trehalose treatment and found i.p. administration of trehalose leads to far greater elevations in serum trehalose relative to oral dosing without observable changes on either atherosclerosis or serum cholesterol (26). Sahebkar et al. similarly showed that i.p. trehalose treatment attenuates atherosclerosis in rabbits (17). Our data also confirmed that i.p. trehalose treatment is therapeutically effective against weight gain and insulin insensitivity, but strikingly that oral-only administration failed to rescue HFD-induced obesity and insulin resistance.

Our results are surprising, because orally administered trehalose has been shown to exert significant biological effects in numerous mouse models of adiposity (19, 21, 22), hepatic steatosis/fat accumulation (23, 24), Alzheimer’s and Parkinson’s diseases (13, 30), and even kidney injury (31). We have compiled a large list of published references detailing the various diseases trehalose has been proposed as a therapeutic strategy and the route of administration for these studies (Table 1). For instance, Arai et al. reported that oral trehalose administration functionally mitigates insulin resistance without affecting body weight (21), but we did not find a similar reduction in insulin resistance, suggesting that oral-only trehalose treatment does not provide a therapeutic benefit under high fat feeding conditions. One possible explanation for this discrepancy is variable experimental conditions, such as diet (e.g., 40% versus 56.7% HFD) or model (e.g., C57BL/6 J mouse versus db/db or ob/ob mouse, or rabbit). However, given that our current and previous data show an almost complete enterohepatic clearance for oral trehalose (26), its bioavailability via parenteral means is likely required to exert clinically important metabolic effects. Moreover, Yasugi et al. suggested that the use of trehalose as a nutrient by organisms was gained via adaption, secondary to trehalose’s ability to contribute to glucose homeostasis by buffering circulating glucose levels, thereby imparting improved metabolism (32). Although this hypothesis is largely based on work done using *Drosophila*, which might not translate to mammals, it would be interesting to know alterations in circulating levels of trehalose in prior studies reporting the metabolic benefits observed with oral trehalose treatment. In all these studies, secondary effects from trehalose on the gastrointestinal tract including effects on gut microbiota may be operative.

**Table 1:** XXX.

Articles	Route	Model
Kaplon et al. (45)	PO	Human
Yoshizane et al. (46)	PO	Human
Yoshizane et al. (47)	PO	Human
Jamialahmadi et al. (48)	IV	Human
Hashemian et al. (49)	PO	Human
Rodríguez-Navarro et al. (50)	PO	Mouse
Castillo et al. (51)	IP	Mouse
Arai et al. (19)	PO	Mouse
Sarkar et al. (52)	PO	Mouse
Kim et al. (53)	IP	Mouse
Ferguson et al. (54)	PO	Mouse
Tanji et al. (55)	PO or IP	Mouse
Sergin et al. (26)	PO + IP	Mouse
Liu et al. (56)	PO	Mouse
Miyake et al. (57)	IP	Mouse
Liu et al. (58)	IP	Mouse
Zhu et al. (31)	IP	Mouse
Gong et al. (59)	PO	Mouse
Pradeloux et al. (60)	PO	Mouse
Yang et al. (61)	PO or PO + IP	Mouse
Lee et al. (62)	PO	Mouse
Forouzanfar et al. (63)	IP	Rat
Wang et al. (64)	PO	Rat

Trehalose is a known nutrient for bacteria and in the could have functional consequences on microbiota which in turn would exert physiological and pathophysiological effects on organisms ingesting trehalose either as part of the diet or as an oral therapeutic. In this regard, contrary to what has been previously reported by others, our data show that metabolic benefits were only observed when trehalose was delivered by parenteral means (i.p. injection) and not by oral administration. Clearly more mechanistic studies are required to determine the underlying basis for trehalose’s modulatory effects on metabolism in mammals, whether previously reported efficacy is dependent on increased blood trehalose levels, and most importantly whether trehalose directly or indirectly influences HFD-induced pathophysiology. Accumulative evidence indicates that trehalose can affect gut microbiota (10, 33, 34), which has been proposed to influence hepatic gene expression (34). This raises the possibility that some of the metabolic benefits attributable to trehalose might therefore be indirect.

It is notable that low serum trehalose levels coupled with high expression of Treh in the intestine would at least theoretically limit the effectiveness of oral treatment versus i.p. administration. One possibility for lack of effect when orally provided is that trehalose is enzymatically cleaved by trehalase in the intestine, where it is highly expressed (35), before entering the bloodstream. This theory is supported by our results showing higher blood glucose levels in mice treated orally with trehalose, compared to those receiving trehalose via i.p. injection (Figure 3). Ishihara and colleagues established a complete cDNA clone of human Treh and showed that Treh mRNA is highly expressed in the kidney, liver and small intestine in humans (36). Similarly, our current findings revealed that Treh is highly expressed in the kidney and duodenum of mice (Figure 4C), results that are identical to what was previously shown by Northern blot (35). Yu et al. reported an association between Treh genetic variance and serum trehalase levels in African Americans and that serum trehalose levels were significantly associated with glucose levels and increased risk of incident diabetes (37). Furthermore, it was recently reported that genetic variants in or near the TREH locus in Pima Indians are strongly associated with trehalase activity, and one of these variants is also reproducibly associated with type 2 diabetes (38).

Kamiya et al. suggested that Treh-deficient mice should be used to observe any physiologically relevant functions of trehalose based on the fact Treh KO abolishes oral trehalose treatment-induced blood glucose without affecting glucose tolerance (39). Our findings indicating that Treh KO boosted the serum trehalose levels via i.p. but not oral treatment (Figure 6) support this idea. However, we failed to find any advanced metabolic benefits due to i.p. trehalose treatment in Treh KO mice (Figure 7), which is similar to what was reported by the Arai group showing that Treh KO failed to boost the anti-obese effect brought by trehalose treatment (40). This evidence implies that systemic Treh is not a clinically appropriate target for treating obesity and related complications, but this does not exclude the possibility of potential metabolic benefits related to tissue-specific suppression of Treh, which should be further studied.

Several unanswered questions remain, particularly the mechanism of action of trehalose in the context of HFD-induced obesity. We previously demonstrate that trehalose triggers autophagy-lysosome biogenesis in macrophages (26) in response to low-grade lysosomal stress, via activation of transcription factor EB (TFEB) (41). This would stimulate a key cellular degradation system including lipid metabolism (via the autophagy of lipid droplets known as lipophagy and lipid hydrolysis in lysosomes). Zhu et al. similarly showed trehalose-mediated induction of TFEB transcriptional activity in a mouse model of acute kidney injury (31). In addition to TFEB activation, trehalose enhances adipocyte lysosomal activity and antioxidative responses via the Sequestosome 1 (SQSTM1/p62)-activated nuclear factor erythroid-derived 2-like 2 (NRF2) (42). Similar findings were described in hepatoma cells, in which trehalose protected against oxidative stress via autophagy, which was regulated by the SQSTM1/p62- Kelch-like ECH-associated protein 1 (KEAP1)-NRF2 pathway (43). Moreover, trehalose can influence autophagy by inhibiting SLC2A family members, such as SLC2A2 and SLC2A8, through AMPK-mammalian target of rapamycin (mTOR) complex 1 (mTORC1) signaling pathway in hepatocytes (23). Although these accumulated reports emphasize an autophagy-dependent role of trehalose in several models, autophagy-independent mechanisms should also be investigated. For example, Pagliassottia et al. showed that trehalose reduces hepatic endoplasmic reticulum stress and inflammatory signaling via restoration of proteasome activity in aged mice (25). Additional mechanistic studies such as these would undoubtedly provide much needed insights into the perceived effects of trehalose on physiologically relevant metabolic homeostasis under basal versus pathological conditions.

Overall, our study shows that trehalose, a natural disaccharide, is a potential candidate for the treatment of HFD-induced obesity, including prevention of insulin resistance and fatty liver, but only when delivered parenterally. In fact, oral trehalose treatment was therapeutically ineffective, and absorption of trehalose could not be improved even in the absence of Treh. Although Treh KO suppressed serum trehalose clearance, it provided no additional anti-obesity benefits or insulin tolerance conferred by i.p. trehalose treatment. Our data provide valuable insight regarding the potential metabolic benefits of trehalose and its effective treatment route, which are especially applicable to its eventual usage clinically, as well as its current use as a supplement for a variety of commercial products.

## Materials and methods

### Animals

Animal protocols were approved by the University of Pittsburgh and Washington University Animal Studies Committees (IACUC 23032631 and 21–0163, respectively). Five-week-old C57BL/6 J male mice were purchased from the Jackson Laboratory (Bar Harbor, ME, United States). Unless otherwise stated, mice were housed at 23 ± 1°C and maintained on a 12-h light/dark cycle. For all experiments, mice were fed a commercial chow diet (Purina 5,053) until 8 weeks of age and then randomly divided into groups for the experiments.

### Strategy and generation of trehalase KO mice

Trehalase (Treh) whole-body knockout mice were established by Genome Engineering &.

Stem Cell Center (McDonnell Genome Institute, Washington University School of Medicine, St. Louis, MO). Briefly, the CRISPR/Cas9 technique was used to target Treh exon 8, and a 13 bp sequence was successfully removed (Figure 5B). Heterozygous Treh knockout (Treh^+/−^) mice were crossed to generate homozygous Treh knockout (Treh^−/−^, Treh KO) mice. Mice without disrupted Treh (Treh^+/+^) were considered as the wildtype (WT) control group. Primer sequences for genotyping were as follows: Forward 5′-AGG ACT GTC TCT GTA GTC TCA GGA GG-3′; Reverse 5′-GAT GCT CAT GTC AGA GTG AGC TGA TGG-3′. Genomic DNA was extracted from mouse tails and amplified using EmeraldAmp PCR Master Mix, followed by visualization by 3% agarose gel electrophoresis (44).

### Mouse phenotype assessment

For examining the influence of trehalose on the metabolic state, mice were fed a chow diet until the indicated age. A diet containing 40% kcal fat (TD 88137, Harlan, United States) was used for all experiments involving high fat diet (HFD) treatment. During HFD feeding, mice were treated with saline, sucrose, or trehalose, by intraperitoneal injection [3 g/Kg body weight (0.3 g trehalose/mL and 10 uL/g body weight), three times per week] and/or by oral route in the drinking water (3% w/v). For trehalose tolerance tests, mice were fasted for 6 h, and a blood sample was taken from the mouse tail vein (0 min point). Subsequently, mice were intraperitoneally treated with a solution of trehalose [3 g/Kg body weight (0.3 g trehalose/mL and 10 uL/g body weight)], and blood samples were collected at indicated time points. For insulin tolerance tests, 15-week HFD-fed mice were fasted for 6 h, blood samples were collected from the mouse tail vein (0 min point) and the mice were then intraperitoneally injected with insulin [1 U/Kg body weight (0.1 U of insulin/ml of sterile PBS, 10 μL/g body weight)]. Mice were pretreated with indicated doses of Validamycin (Cayman Chemicals, Ann Arbor, MI, United States) intraperitoneally, followed by trehalose (3 g/Kg body weight), 3 h before assessment of blood glucose levels. Blood samples were obtained at indicated time points. Body composition was determined by EchoMRI-100H (EchoMRI LLC) and presented as fat and lean mass, in accordance with the manufacturer’s instructions. For tissue *Treh* expression profiles and for tissue Treh activity assays, tissues were harvested from 8-week-old male C57BL/6 J mice.

### Histological analysis and tissue weights

BAT, iWAT, eWAT, and liver were weighed upon mouse dissection. Liver tissue samples were fixed in 4% paraformaldehyde overnight and then transferred to 70% ethanol. The fixed samples were embedded in paraffin for staining with hematoxylin and eosin Y.

### Analysis of plasma parameters

Glucose, cholesterol, TGs, and FFA concentrations were determined using commercially available kits: Autokit Glucose (NC9927772, Wako Chemicals, Richmond, VA, United States), Infinity Cholesterol Liquid Stable Reagent (TR13421, Thermo Fisher), Infinity Triglyceride Reagent (TR22421, Thermo Fisher), and NEFA HR[2; Reagent 1 (434–91,795) and Reagent 2 (436–91,995), Wako Chemicals], respectively. All kits were used in accordance with the manufacturer’s instructions.

### Analysis of trehalose and trehalase activity

Trehalose concentration and trehalase activity were measured using Trehalose Assay Kit (Megazyme, Bray, Ireland) according to manufacturer protocol. Briefly, tissue homogenate (equal protein concentrations between samples) or plasma was treated with an equal volume of 5 g/L trehalose and then incubated at 37°C for 30 min. After that, the plate was read at 340 nm every 5 min or until values stop changing.

### Gene expression quantification

RNA was isolated with Ambion PureLink RNA Kit (Life Technologies, Foster City, CA) and then reverse-transcribed using SuperScript VILO (Life Technologies). For quantification of mRNA expression, real-time PCR was performed with a LightCycler system (QuantStudio; Thermo Fisher, Waltham, MA) using SYBR Green fluorescence signals (SYBR Select Master Mix, 4,472,908, Thermo Fisher). The protocol for amplification was as follows: denaturation, 95°C for 1 min; annealing, 60°C for 10 s; extension, 72°C for 40 s. Gene expression levels were normalized to the levels of ribosomal protein lateral stalk subunit P0 (Rplp0) Forward 5′- ATC CCT GAC GCA CCG CCG TGA-3′; Reverse 5′- TGC ATC TGC TTG GAG CCC ACG TT-3′. Primer sequences used for *Treh*: Forward 5’-TCA TCT TGG TAG AGC TGG GC-3′; Reverse 5′- ATG ACC TGG GAG CTG CAC-3′.

### Western blotting

Cells or tissues were lysed in a standard RIPA lysis buffer. Standard techniques were used for protein quantification, separation, transfer, and blotting. The following primary antibody was used: Treh (1:1000, sc-390034, Santa Cruz, Heidelberg, Germany). Ponceau-S total protein staining was used as a loading control.

### Quantification and statistical analysis

All results are presented as mean ± SEM. Bars in graphs represent standard errors, and significance was assessed by two-tailed Student’s *t* tests.

## Data Availability

The datasets presented in this article are not readily available because no dataset used in the current manuscript. Requests to access the datasets should be directed to brazani@pitt.edu.
