# Supplementary material for: Assessing the efficacy of the natural disaccharide trehalose in ameliorating diet-induced obesity and metabolic dysfunction
Source: Front Nutr. 2025 Jun 2;12:1580684. doi: 10.3389/fnut.2025.1580684 (PMC12171462; doi:10.3389/fnut.2025.1580684)
Supplement: Supplementary file 1 [file Data_Sheet_1.pdf]

Figure S1

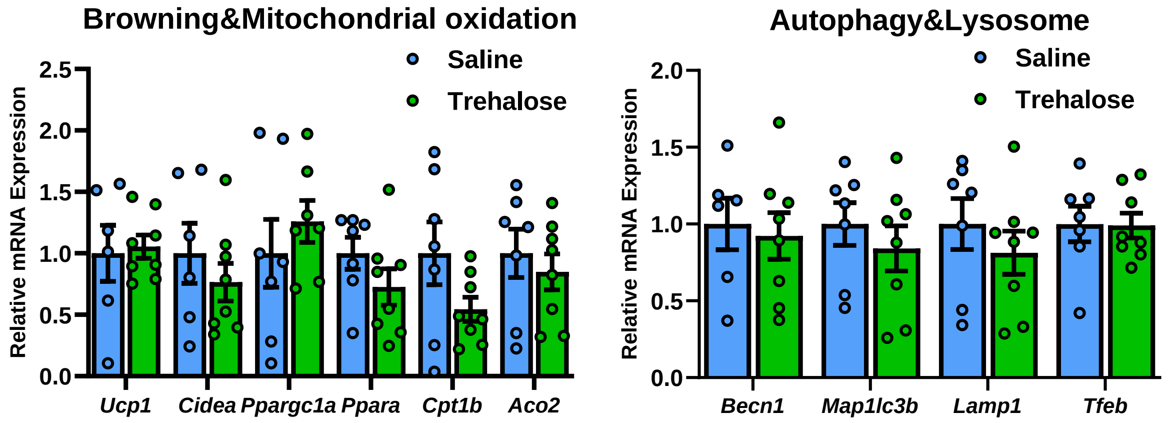

Figure S1. Trehalose Did Not Affect Gene Expression in Adipose Tissue

Gene expression levels of browning, mitochondrial oxidation, and autophagy/lysosome-related genes were measured in inguinal white adipose tissue (n = 6–8) after 16 weeks of high-fat diet and combined oral and intraperitoneal trehalose treatment. All mice were male. Values are presented as mean ± SE.
